# P-1137. Decreasing Empiric Vancomycin use for Orbital Cellulitis in Children

**DOI:** 10.1093/ofid/ofae631.1324

**Published:** 2025-01-29

**Authors:** Rachel Downey, Nisha Parihar, Natalie Weston, Soman Khan, Sarmistha Bhaduri. Hauger, Alan Wade Mincher, Travis Kozak

**Affiliations:** Dell Children's Medical Center of Central Texas, Austin, Texas; University of Texas at Austin, Austin, Texas; Dell Medical School at the University of Texas, Austin, Texas; Dell Medical School, Austin, Texas; Dell Children's Medical Center; Dell Medical School at the University of Texas at Austin, Austin, Texas; Dell Children's Medical Center of Central Texas; Dell Medical School at UT Austin, Austin, Texas; Vanderbilt University, Nashville, Tennessee

## Abstract

**Background:**

Orbital cellulitis in children is often caused by *Staphylococcus aureus, Streptococcus viridans* group, and *Streptococcus pyogenes*. Oral, respiratory, and skin commensals are also considered in empiric antibiotic choice. MRSA is becoming less prevalent nationally.

In 2015, our hospital implemented an evidence-based orbital cellulitis guideline to standardize care and promote antibiotic and surgical stewardship. The guidance calls for empiric clindamycin and ceftriaxone, reserving vancomycin for cases with signs of optic nerve or CNS involvement. Here we evaluate results of this guidance and summarize isolated bacteria.

**Fig 1.**
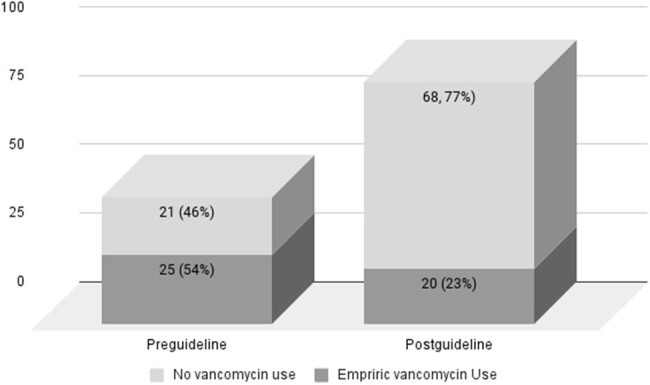
Empiric use of vancomycin pre and post guideline

**Methods:**

Cases were pulled from the hospital chart by diagnosis code and positive computed tomography scan for orbital cellulitis for 4 years pre-guideline and (2009-2013) 7 years post-guideline (2015-2021) implementation and were manually reviewed for clinical diagnosis of orbital cellulitis. Variables collected included demographics, empiric antibiotics, presence of abscess, surgical intervention, bacterial identification by blood or surgical culture, length of stay, and related readmission. Statistic analysis included chi square, fisher's exact test and Wilcoxon ranked sum.

**Fig 2.**
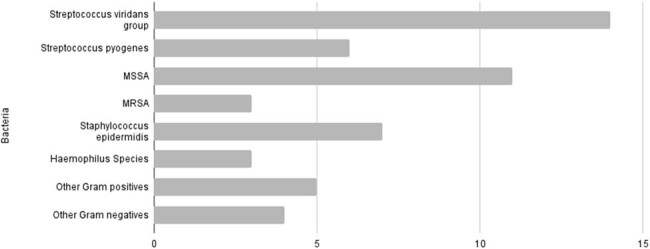
Bacteria isolated from blood or surgical culture in all cases *13 cases were polymicrobial

**Results:**

134 cases were included in the analysis (46 pre and 88 post guideline). Median age was 7 in both groups; there was incidentally a significantly higher proportion of males in the post-guideline group (54% v 75%, p=0.025). Post guideline, empiric use of vancomycin significantly decreased from 54% to 23% (p< 0.001) (fig 1). Among cases with vancomycin use post-guidance, 60% of use was concordant with the guideline. Length of stay was reduced from a median of 4 days to 3 days (p=0.001). Rate of abscesses and surgical intervention were nearly identical in pre and post populations (63% with abscess, and 24% with surgery in both groups). Overall, bacterial cultures from blood or abscess were positive in 26% of cases (10 (22%) pre, 25 (28%) post guideline); 13 (37%) were polymicrobial. All 3 MRSA cases were susceptible to clindamycin.

**Conclusion:**

Implementation of a hospital-wide guideline can result in more judicious use of broad spectrum antibiotics. Reduction in vancomycin occurred without increased length of stay, need for surgical intervention, or readmission.

**Disclosures:**

**All Authors**: No reported disclosures

